# Heat shock proteins in the physiology and pathophysiology of epidermal keratinocytes

**DOI:** 10.1007/s12192-019-01044-5

**Published:** 2019-11-16

**Authors:** Dorota Scieglinska, Zdzisław Krawczyk, Damian Robert Sojka, Agnieszka Gogler-Pigłowska

**Affiliations:** Center for Translational Research and Molecular Biology of Cancer, Maria Skłodowska-Curie Institute-Oncology Center Gliwice Branch, ul. Wybrzeże Armii Krajowej 15, 44-101 Gliwice, Poland

**Keywords:** Heat shock proteins, Epidermis, Keratinocytes, Cytoprotection, Epidermal homeostasis, Keratinocyte differentiation

## Abstract

Heat shock proteins (HSPs), a large group of highly evolutionary conserved proteins, are considered to be main elements of the cellular proteoprotection system. HSPs are encoded by genes activated during the exposure of cells to proteotoxic factors, as well as by genes that are expressed constitutively under physiological conditions. HSPs, having properties of molecular chaperones, are involved in controlling/modulation of multiple cellular and physiological processes. In the presented review, we summarize the current knowledge on HSPs in the biology of epidermis, the outer skin layer composed of stratified squamous epithelium. This tissue has a vital barrier function preventing from dehydratation due to passive diffusion of water out of the skin, and protecting from infection and other environmental insults. We focused on HSPB1 (HSP27), HSPA1 (HSP70), HSPA2, and HSPC (HSP90), because only these HSPs have been studied in the context of physiology and pathophysiology of the epidermis. The analysis of literature data shows that HSPB1 plays a role in the regulation of final steps of keratinization; HSPA1 is involved in the cytoprotection, HSPA2 contributes to the early steps of keratinocyte differentiation, while HSPC is essential in the re-epithelialization process. Since HSPs have diverse functions in various types of somatic tissues, in spite of multiple investigations, open questions still remain about detailed roles of a particular HSP isoform in the biology of epidermal keratinocytes.

## Introduction

Heat shock proteins (HSPs) are principally referred to as molecular chaperones. Having diverse intracellular localization and functional differentiation, HSPs are involved in folding newly synthesized proteins, assembly of protein complexes, intracellular transport of proteins, or prevention of denaturation and/or unspecific aggregation of improperly folded proteins both under physiological and stress conditions. Through chaperoning multiple client proteins, HSPs participate in modulation of signal transduction including immunological responses, while by cooperation with the ubiquitination system, participate in directing irreversibly damaged proteins into proteasomal degradation (Saibil 2013; Palotai et al. 2008). The research on HSPs in epidermal biology has been focused on investigating their functions in the context of physiological processes and also in response to different environmental stress conditions. It is noteworthy that epidermal keratinocytes, in contrast to other somatic cells, are unique in terms of expressing significant amounts of inducible HSPs constitutively, without previous stress challenge.

On the basis of molecular weight and amino acid (aa) sequence similarity, HSPs are subdivided into several families, namely HSPB (small HSP), HSPA (HSP70), DNAJ (HSP40), HSPC (HSP90), and HSPH (HSP110). The first name in the above list refers to the novel nomenclature system (Kampinga et al. 2009), which eliminates ambiguity in HSPs naming accepted by the HUGO Gene Nomenclature Committee (HGNC); the denotation in parenthesis conforms to common/historical names of the major HSP families (Lindquist and Craig 1988; Tóth et al. 2015).

The epidermis, the outermost layer of the vertebrate skin, is a squamous, stratified, non-vascularized cornified epithelium of ectodermal origin. This multilayered epithelium provides a barrier between the body and external environment, protecting mammals from dehydration, infection and environmental toxins. The major cellular component of epidermis are keratinocytes (around 90–95% of the epidermal cell population), which undergo a tightly controlled program of terminal differentiation. During this process, the undifferentiated keratinocytes from the basal layer (the innermost layer of the epidermis) permanently withdraw from the cell cycle, change their biological activity and morphology, and move suprabasally to form *stratumspinosum*, *stratumgranulosum* and *stratumcorneum*. The outermost epidermal layer consists of dead, flattened, non-nucleated keratinocytes termed corneocytes. This is the essential structure of the epidermal barrier.

**Fig. 1:**
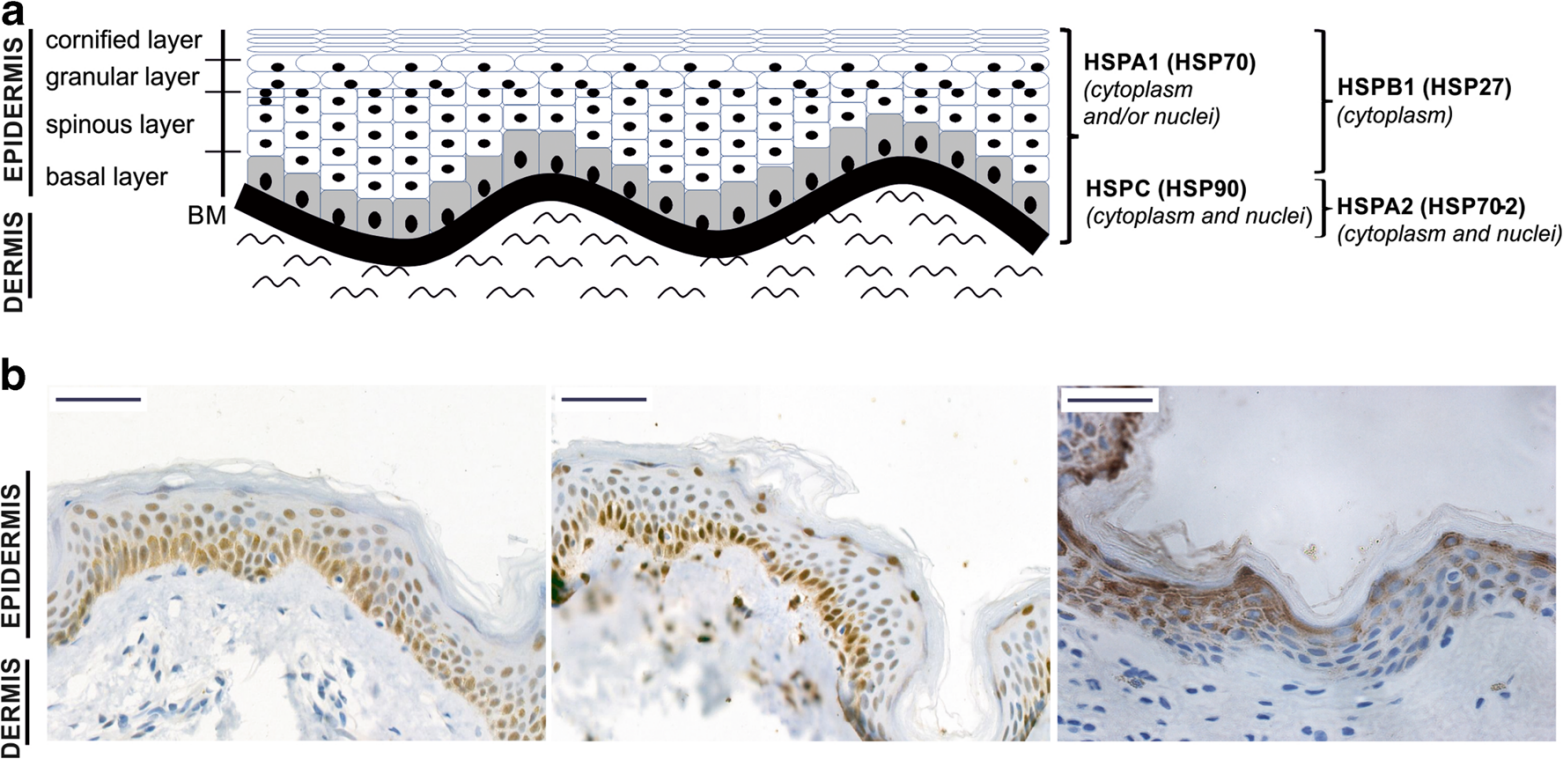
Differential expression of HSPs in human epidermis. **a** Schematic representation of HSPC (HSP90), HSPA1 (HSP70), HSPA2, and HSPB1 (HSP27) location based on the results of numerous works cited in this review article. **b** Location of the HSPB1, HSPA1, and HSPA2 proteins in human skin. Microphotographs showing examples of DAB-mediated immunohistochemical staining in formalin-fixed and paraffin-embedded human skin. The skin sections were incubated overnight with primary antibodies: mouse monoclonal anti-HSPB1 (Novocastra, 1:40); mouse monoclonal anti-HSPA1 (Enzo Life Science; 1:300); rabbit monoclonal anti-HSPA2 (Abcam, 1:6000). The bar represents 50 μM

Human epidermis follows a tightly regulated proliferation and differentiation program with an interplay between stem cell self-renewal and differentiation in order to maintain tissue homeostasis (Fig. 1). Despite decades of skin research, regulation of homeostasis in human epidermis is still insufficiently understood and remains a matter of debate. In particular, the data are insufficient to convincingly determine the cell dynamic in interfollicular epidermis in human skin. Significant amount of data comes from studies conducted on the interfollicular epidermis of mouse skin. Despite evident differences between mouse and human epidermis with respect to stratification and thickness, it is believed that the basic maintenance mechanism of epidermal homeostasis might be quite similar in human and mouse, as many conclusions derived from studying epithelial biology in mouse or rat models are considered relevant also to human tissue. Since the work of Potten (1974), the long-held concept of epidermal proliferative units (EPUs) postulated that self-renewal of the epidermis is dependent on slow-cycling, long-lived, and self-renewing keratinocyte stem cells which constitute 1–10% cells of the basal layer and undergo asymmetric divisions giving rise to one long-lived stem cell and the other, short-living one called transit amplifying cell (TA). TA cells were supposed to divide mitotically several times before undergoing terminal differentiation. The model postulated that epidermis is composed of EPU each having a columnar shape perpendicularly oriented from the basal membrane to the top of the cornified layer and containing basally located one stem cell surrounded by approximately ten TA cells and their maturing progeny located suprabasally (Strachan and Ghadially 2008; Senoo 2013).

Advances in cell tracing analysis caused the EPU model to be questioned, and currently, alternative ideas were proposed to explain the maintenance of epidermal homeostasis (reviewed in Gonzalez-Celeiro et al. 2016). Among them, the hierarchical model assumes that the basal layer contains slow-cycling long-term stem cells (SCs) that in turn give rise to fast-dividing cells termed “committed progenitors.” Another concept, called a regional specificity model, postulates that slow-cycling and fast-cycling stem cells co-exist, but they occupy and maintain different domains in the epidermis. The last but not least stochastic choice model assumes that all basal cells are equivalent in terms of their potential to divide or differentiate. The critical belief in all these models is that the progenitor cells divide asymmetrically. This mechanism allows for generation of sibling cells that adopt distinct fate decision—one of them remains progenitor cell while the other one is committed into differentiation. Alternatively, symmetric division of progenitors can also give rise either to two progenitors or two post-mitotic differentiating cells; however, such scenarios are likely to happen less frequently than asymmetric divisions (Gonzalez-Celeiro et al. 2016). Thus, it is postulated that the homeostasis of interfolicular epidermis is maintained by fast-dividing progenitor cells. In turn, a small fraction of slow-cycling multipotent/pluripotent stem cells, which is clustered around the hair follicles, or placed under the edges of the overlying scales in the hairless skin of rodents tail, is likely responsible for the wound healing and regenerative processes, but make marginal contribution to epidermal homeostasis (Doupe and Jones 2012; Alcolea and Jones 2014; Mascré et al. 2012). Essentially, it seems accepted that the basal layer of the interfolicular epidermis has heterogeneous composition and contains progenitors cells, post-mitotic cells, and a small fraction of stem cells.

Keratinocytes are also responsible for restoring the epidermis after cutaneous injury. Re-epithelialization, one of the early steps of multistage wound healing process, is a temporal reprogramming of the phenotype of keratinocytes in the basal layer toward migratory state characterized by high ability to migrate laterally into the wound over the denuded area. This phenotypic switch manifests by a loss of the extracellular matrix-cell and cell-cell contacts and acquisition of capability to interact with a provisional extracellular matrix formed by proteins of the clot. During migration, keratinocytes restrain their proliferation, albeit divisions can be restored when the first layer of keratinocytes covers the wound (Gonzalez et al. 2016). These tightly coordinated processes ensure epidermal homeostasis re-establishment of injured skin. Keratinocyte proliferation and migration are crucial steps for the rapid closure of the epidermis during wound healing, but the molecular mechanisms involved in this cellular response remain to be completely elucidated.

Considering pleiotropic roles of HSPs, both under physiological and stress conditions, in this review, we summarized current knowledge of HSPA1 (HSP70), HSPA2, HSPB1 (HSP27), and HSPC (HSP90) in keratinocyte biology. Of all HSPs, the above-mentioned proteins have been the most intensively investigated ones in the context of epidermal homeostasis. Here, we discuss their involvement in keratinocyte cytoprotection, epidermal differentiation, and wound healing. Some aspects concerning implications of HSPs in plastic surgery, dermatology, skin aging, and immunotherapy of skin cancer have been discussed in earlier reviews (Morris 2002; Jonak et al. 2006; Wagstaff et al. 2007; Vidal Magalhaes et al. 2012).

## Essentials about the HSPB1 protein, a member of the HSPB multigene family encoding low molecular weight (12–43 kDa) HSP proteins

The HSPB family contains several members of which some are expressed ubiquitously, and others in a cell-type specific fashion (Arrigo 2017). Among several members of the human HSPB family, only HSPB1 (historical names: HSP27 in human, HSP25 in rodents) has been investigated in the context of keratinocyte biology. HSPB1 is a stress-inducible chaperone, which having so called holdase activity participates in transferring unfolded peptides to HSPA-HSPC-HSPH chaperone machinery supporting them to achieve native structures and preventing from unspecific aggregation (reviewed in: Hilton et al. 2013; Treweek et al. 2014). Structural modifications of HSPB1 that comprise oligomerization and specific phosphorylation pattern are considered critical for regulating its interaction with specific clients and shaping its cell type-and condition-specific functions (Arrigo 2013). Under cellular stress, some protein pool of HSPB1 migrates from cytoplasm into the nucleus (Bryantsev et al. 2007), and/or can be secreted outside the cell (Pockley et al. 2014). HSPB1, considered one of the most important elements of cellular proteostasis, is functionally involved in numerous physiological processes and can be also engaged in sustaining pathological states (Kampinga and Garrido 2012). HSPB1 is usually overexpressed in various tumors and cancer cell lines. Depending on the tumor type, its elevated expression was associated with either good or bad prognosis, or did not show prognostic value (Kaigorodova and Bogatyuk 2014).

### Expression of HSPB1 in normal epidermis and pathological epidermal lesions

Presence of the HSPB1 protein in keratinocytes positioned in the suprabasal layers of healthy human epidermis was revealed by immunohistochemical (IHC) studies (Gandour-Edwards et al. 1994; Trautinger et al. 1995a). Similar expression pattern was observed in normal murine epidermis, albeit Laplante et al. (1998) noted absence of HSPB1 in *stratum corneum*, while Crowe et al. (2013) showed its preferential location in this layer. Selective or predominant presence of HSPB1 in upper epidermal layers, specifically in the granular one, was confirmed in tissue-engineered skin reconstructed from either human or rat keratinocytes (Robitaille et al. 2010; Jonak et al. 2011; O'Shaughnessy et al. 2007). Co-localization studies revealed that in terminally differentiated keratinocytes, HSPB1 was a component of peripherally located multiprotein complexes comprising among others loricrin and filaggrin, key proteins engaged in the cornification process (Jonak et al. 2002). IHC studies showed that transient heat shock (HS) induced both HSPB1 expression and its translocation from cytoplasm into the nucleus in normal human epidermal keratinocytes (NHEK) and HaCaT cells in vitro (McClaren and Isseroff 1994), and in locally heated areas of human buttock skin in vivo (Wilson et al. 2000). Interestingly, HSPB1 expression was also shown to be activated in human skin explants by freezing stress (Jeanmaire et al. 2003).

The expression of HSPB1 was also analyzed in developing epidermis during embryonal development in human and mice (Jantschitsch et al. 1998; Monastirli et al. 2005; Duverger and Morange 2005; O'Shaughnessy et al. 2007). Comparing HSPB1 expression in the embryonal epidermis of mouse and human, one may notice that in the latter case, this protein is absent in the single-layered primitive epidermis and its synthesis starts along with activation of differentiation process. Also, HSPB1 shows uniform cytoplasmic distribution in human keratinocytes, while in murine cells, it localizes in cytoplasmic spots.

Altered expression of HSPB1 was observed in various pathological skin conditions. Its decreased level was detected in cutaneous squamous cell carcinoma (SCC) (Gandour-Edwards et al. 1994; Trautinger et al. 1995a), in UV-induced murine skin SCC tumors (Kiriyama et al. 2001), and in human epidermoid carcinoma A431 cells (Kindas-Mugge et al. 1996). Significant reduction in HSPB1 expression was observed in some rare skin diseases such as bullous ichthyosiform erythroderma and annular epidermolytic ichthyosis, and was not detected in keratinocytes derived from lesional skin of some patients with X-linked recessive ichthyosis, congenital hemidysplasia with ichthyosiform nevus, and limb defects syndrome. In turn, the pattern of HSPB1 expression was similar to healthy skin in other than above-mentioned hereditary cases of ichthyosis (Jonak et al. 2005), as well as in non-cancerous skin lesions such as actinic keratosis, seborrhoeic keratosis, solar keratosis, and human papillomavirus-induced keratinocyte hyperproliferation (Trautinger et al. 1995a; Gandour-Edwards et al. 1994). In contrast, keratinocytes in inflammatory skin diseases such as psoriasis (Boehncke et al. 1994; Choi et al. 2012) and atopic dermatitis (AD) (Niiyama et al. 2016) were found to contain significantly higher levels of HSPB1 than keratinocytes of healthy skin. However, no essential changes in HSPB1 expression between keratinocytes of AD and healthy skin were reported by others (Ghoreishi 2000).

Interesting link between HSPB1 and psoriasis emerged from studies aimed at analyzing the phenotype of cells deficient in the DNAJA3 (TID) protein, one of the members of numerous DNAJ (HSP40) family. It was found that TID-1s, the shortest splicing form of DNAJA3, when complexed with MK5 kinase, inhibited HSPB1 phosphorylation and negatively affected polymerization of F-actin in HeLa cells. Oppositely to HeLa cells, psoriatic keratinocytes were negative for TID-1s and contained increased levels of phosphorylated HSPB1. In HaCaT cells, increasing TID-1s levels interfered with TNF-α induced phosphorylation of HSPB1 and polymerization of F-actin, as well as inhibited TNF-stimulated cell migration. These results suggested that the loss of the TID-1s is one of factors responsible for the development of psoriasis via mechanism partially related to enhanced phosphorylation of HSPB1 (Choi et al. 2012). Another study suggested HSPB1 to be among autoantigens of a streptococcal-induced autoimmune response, being one of the targets of the exaggerated T cell response in psoriasis (Besgen et al. 2010).

### The role of HSPB1 in regulating keratinocyte differentiation in adult epidermis

The pattern of HSPB1 expression in epidermis implied participation of this chaperone in the late stages of keratinocyte differentiation. One of well-known regulator of keratinocyte differentiation is calcium ions (Ca^2+^) (Elsholz et al. 2014). Low Ca^2+^ levels (0.03–0.05 mM) in media maintain basal-like phenotype of keratinocytes, while higher Ca^2+^ levels (0.5–1.2 mM) activate differentiation process. Early studies showed that HSPB1 was barely detectable in NHEK grown in low Ca^2+^ media, whereas a switch to high Ca^2+^ concentration significantly raised the intracellular level of HSPB1 and increased subpopulation of HSPB1-positive keratinocytes (Kindas-Mugge and Trautinger 1994**)**. Ca^2+^-mediated increase in HSPB1 expression was confirmed in studies performed on human HaCaT (Arrigo and Ducasse 2002), and murine PAM212 cells (Duverger et al. 2004). Increased levels of HSPB1 in NHEK were also found during differentiation induced in vitro by high cell confluency, or treatment with calcium ionophore A23187, as well as during formation of granular layer in reconstructed epidermis (Robitaille et al. 2010).

HSPB1 underdoes phosphorylation and forms aggregates in the cytoplasm and nuclei of differentiating keratinocytes. The p38 mitogen-activated protein kinases (p38 MAPK) signaling cascade represent one of pathways engaged in HSPB1 phosphorylation (Duverger et al. 2004; Jonak et al. 2011). Inhibition of p38-MAPK signaling prevented Ca^2+^-induced terminal differentiation of NHEK simultaneously blocking phosphorylation of HSPB1. Similarly, inhibition of p38-MAPK paralleled the phenotypic effects of RNAi-mediated knockdown of HSPB1 in reconstructed epidermis model; both treatments caused aberrant stratification. Thus, p38-MAPK-mediated phosphorylation of HSPB1 is vital for keratinocyte differentiation and formation of regularly stratified epidermis (Jonak et al. 2011).

In keratinocytes, HSPB1 can undergo phosphorylation also by Dual Leucine zipper-bearing Kinase (DLK), an upstream activator of the MAP kinase pathways (Robitaille et al. 2005). Adenoviral-mediated overexpression of DLK in NHEK caused relocation of a fraction of HSPB1 into insoluble cytoskeletal complexes during formation of cornified envelope, but had no effect on a total level of the protein. In turn, siRNA-mediated knockdown of HSPB1 in DLK-overexpressing HaCaT cells interfered with involucrin expression, suggesting a potential role for HSPB1 and DLK in regulating involucrin stability and/or expression. It seems that DLK affects functional properties of HSPB1 not by itself, but rather via activation of ERK kinase responsible for phosphorylation and cytoskeletal redistribution of HSPB1. DLK also activates JNK pathway capable to counteract HSPB1 phosphorylation by reducing ERK activity (Robitaille et al. 2010). Recently, RHE model-based study revealed that DLK1 promotes peripheral localization of HSPB1, as well as LIS1, a protein involved in cortical organization of microtubules in differentiating keratinocytes (Simard-Bisson et al. 2017).

Yet, another kinase known to play an essential role in regulating HSPB1 functionality is AKT. Double knockout of *AKT1* and *AKT2* genes in mouse caused early postnatal lethality and a serious cutaneous defect manifested by a lack of *stratum corneum*. In contrast, mice with single knockdown of either *AKT1* or *AKT2* were viable and had phenotypically normal skin showing only subtle disturbances in the formation of cornified envelope. Their epidermis contained significantly reduced levels of phosphorylated HSPB1, what suggested that both kinases contribute to posttranslational modification of this chaperone in keratinocytes. What is more, AKT1-dependent phosphorylation of HSPB1 seems to promote its binding to filaggrin, filaggrin maturation, and development of *stratum corneum* (O'Shaughnessy et al. 2007). Further study showed AKT1 activity to be important for switching HSPB1 function from actin stabilization to filaggrin processing (Gutowska-Owsiak et al. 2018). Altogether, the above results indicated that AKT1-dependent modulation of HSPB1 activity may be vital for cornification and formation of a fully functional skin barrier. Surprisingly, study of HSPB1^del/del^ mice showed that HSPB1 is dispensable for normal development and maintenance of the unwounded epidermis in vivo (Huang et al. 2007; Crowe et al. 2013). It turned out, however, that HSPB1 is required for wound healing process since the phenotypic alterations in knockout mice manifested after skin wounding and comprised reduced re-epithelialization and increased inflammation (Crowe et al. 2013).

### The influence of UV light and chemical irritants on HSPB1 expression in keratinocytes

Epidermal keratinocytes, being frequently exposed to elevated temperature, are also commonly subjected to sun’s ultraviolet radiation (UV) which consists mostly (96–99%) of long wave ultraviolet (UVA; 320–400 nm), and to less extent (1–6%) of short wave ultraviolet (UVB; 290–320 nm). While UVA can reach dermis, UVB is almost completely absorbed by the epidermis, and constitutes a main environmental factor damaging keratinocyte DNA. UVC (100–290 nm), the third component of solar radiation, is entirely absorbed by the atmosphere; thus, no significant irradiation of the skin results from natural sources. Most harmful effect of phototoxicity is a development of skin cancer (reviewed in: D’Orazio et al. 2013; Kim et al. 2015).

Transcriptomic studies indicated HSPB1 mRNA as one of seven protein coding sequences, expression of which increased at least threefold after exposure of human keratinocytes to UVB in vitro (Becker et al. 2001). UV-induced expression of HSPB1 was also observed in NHEK cells irradiated with the UVB dose equivalent to sun exposure causing mild skin redness in people with light complexion (Wong et al. 2000), and in human skin ex vivo model exposed to radiation mimicking solar light (Jeanmaire et al. 2003). Irradiation of dorsal skin of female hairless mice or PAM212 keratinocytes with physiologically relevant doses of UVB induced nuclear and/or perinuclear accumulation of HSPB1 and stimulated its phosphorylation (Nozaki et al. 1997). Similar pattern was observed in human keratinocytes, and in this case, UVB-induced phosphorylation of HSPB1 was executed by p38 MAPK signaling cascade possibly via generation of reactive oxygen species (Wong et al. 2000). Studies performed on telomerase-immortalized keratinocytes revealed that solar UV or equivalent dose of UVB significantly increased the level of phosphorylated HSPB1 and led to activation of p38α and MSK2 kinases, at the same time decreasing the activity of ERK kinases and having minimal impact on several other variants of p38 kinase (p38ß, p38γ and p38δ). In contrast, UVA had minimal effect on both HSPB1 phosphorylation and activity of kinase signaling pathways. These results confirmed that the key signaling pathway activated by both solar and UVB radiation is dependent on p38α kinase activity, and that this pathway plays a key role in HSPB1 phosphorylation (Liu et al. 2013).

Because harmful environmental hazards such as HS or UV have a clear impact on the HSPB1 expression and its posttranslational modifications, a number of studies were undertaken to assess whether HSPB1 could be useful as a marker for identification of skin irritants related to occupational or environmental exposure, or present in cosmetic products. Given that due to EU regulations, cosmetic testing in animal models is banned, the toxicity tests are conducted in vitro, on keratinocytes growing either in 2D monolayer or 3D organotypic culture. Proteomic analysis revealed that HSPB1 is among several proteins upregulated in human epidermis (Boxman et al. 2002b; Chen et al. 2014), or primary foreskin keratinocytes (Zhang et al. 2011) exposed to sodium lauryl sulfate (SLS), a surfactant widely used for inducing the experimental contact dermatitis. Increase in HSPB1 expression was also reported in HaCaT cells exposed to heavy metals (potassium dichromate, neodymium nitrate), or sodium dodecyl sulfate (SDS), contaminants of cosmetic products that may cause inflammatory skin conditions such as eczema, lichenoid, or vasculitis (Zhang et al. 2010). Higher levels of HSPB1 were found in keratinocytes exposed to mustard gas or its derivatives (2-chloroethyl ethyl sulfide; CEES) (Mol et al. 2008; Black et al. 2011), to ozone (O_3_) (Valacchi et al. 2002, 2003), and to exhaust gas or cigarette smoke (Jeanmaire et al. 2003). Also parabens, a class of preserving additives found in cosmetics that can cause skin irritation and contact dermatitis, elevated the levels of HSPB1 in skin equivalents (Ishiwatari et al. 2007). Similar findings were reported in the epidermis of bitumen exposed workers, and notably, HSPB1 was found in all epidermal layers including the basal one (Fenga et al. 2000). Interestingly, HSPB1 expression was not changed by mechanical skin irritation caused by type stripping; this observation may indicate that HSPB1 should not be regarded as a universal marker of skin irritation (Dickel et al. 2010).

### HSPB1 as cytoprotective and immunomodulatory factor

HSPB1 is classified as a molecular chaperone; thus, one could assume that its increased expression in keratinocytes exposed to cytotoxic factors may reflect activation of an adaptive response. Systematic study that would unambiguously demonstrate protective function of HSPB1, however, has not been performed so far. Some premise was provided by study on A431 skin cancer cells transfected with vector encoding the human *HSPB1* gene. After transfection, the cells became slightly more resistant to HS, but their sensitivity to UVA, UVB, or hydrogen peroxide remained unaffected (Trautinger et al. 1997). Recent studies on mice showed that the inactivation of HSPB1 (as well as HSPA1) in vivo by locally administrated antibodies reduced the incidence of neoplastic lesions in the skin treated with 7,12-dimethylbenzene(a)anthracene (DMBA) and benzo(a)pyrene (Yusuf et al. 2015). It is worth noting that the translocation of HSPB1 into the keratinocytes nuclei following stress (treatment with SLS or UV) can be modulated by antioxidants (vitamin C) (Boxman et al. 2002a).

HSPB1 may also play a role in regulating inflammatory pathways in the epidermis. The depletion of HSPB1 in keratinocytes through siRNA-mediated gene silencing stimulated production of PGE2 both in the control and TNF-α-activated cells, as well as release of IL-1α, IL-8 upon UVB irradiation or TNF-α stimuli. Moreover, HSPB1 can differentially influence synthesis of proinflammatory cytokines in keratinocytes and dermal fibroblasts (DFs)—suppressing the process in keratinocytes while activating in DFs (Sur et al. 2008).

## Essentials about the HSPA1, a member of the conservative HSPA (HSP70) family

The HSPA1 is one of the most thoroughly investigated HS-inducible chaperone (Radons 2016). The human genome contains two intronless *HSPA1* genes localized within the MHC III region, namely *HSPA1A* and *HSPA1B,* which share 99% nucleotide sequence similarity in the protein coding region. Encoded proteins differ only by two aa, thus are considered functionally equal. Generally, HSPA1 is weakly expressed (if at all) in somatic cells under physiological conditions, but its levels can be highly elevated by HS and other environmental stresses as well as under various pathological conditions, particularly in tumors (Calderwood 2018). However, it is known that HSPA1 can be produced constitutively at relatively high levels in certain somatic cell types (Scieglinska et al. 2011). The stress-induced expression of HSPA1 is a transcriptional response initiated via binding of a heat shock transcription factor 1 (HSF1) to the promoter regulatory sequence called heat shock element (HSE). Upon stress a significant fraction of HSPA1, protein translocates from the cytoplasm into the nucleus and nucleoli (Pelham 1984). HSPA1, in cooperation with several other HSPs and co-chaperones, plays a critical cytoprotective role enabling survival of stressed cells (Radons 2016). Molecular stresses or pathophysiological conditions can also stimulate relocation of HSPA1 into extracellular space or/and onto the plasma membrane. Such responses contribute to the modulation of immune processes (both innate and adaptive) and inflammation (Multhoff and Hightower 2011; Calderwood et al. 2016).

The general features of HSPA1 are relatively well established; however, there are some problems in analyzing the literature data, namely inconsistency in the HSPAs nomenclature, and discrepancies related to results of HSPA1 immunodetection due to poor specificity of anti-HSPA1 antibodies. The second issue seems extremely disturbing because it is well known that even frequently used commercial antibodies may cross-react with different highly homologous members of the HSPA family (Malusecka et al. 2006; Chow et al. 2010; Scieglinska et al. 2011).

### Expression of HSPA1 in the normal skin

The earliest studies based on radioactive protein-labelling techniques (Deaton et al. 1990; Edwards et al. 1991; Maytin et al. 1990), as well as further research performed using more advanced methodology (e.g., IHC, western blotting, mass spectroscopy), revealed that at physiological temperature, the stress-inducible HSPA1 protein is constitutively present in isolated keratinocytes and HaCaT cells, and it localizes mainly in the cytoplasm (Schmidt-Rose et al. 1999; Lamore et al. 2010; Perluigi et al. 2010; Parat et al. 1998; Evdonin et al. 2006; Nakamura et al. 2010; Prado et al. 2011; Hintzsche et al. 2012; Ramirez et al. 2015).

Initial IHC studies performed on human skin explants generated inconsistent results showing either no HSPA1-positive reaction both in the epidermis and dermis (Muramatsu et al. 1992), low intensity staining in epidermal keratinocytes (Wilson et al. 2000; Boyman et al. 2005), or very strong staining selectively in the epidermis (Trautinger et al. 1993; Curry et al. 2003). These discrepancies in the expression pattern are thought to result from the use of anti-HSPA1 antibodies of poor or unverified specificity. Most recent IHC studies that ensured satisfactory specificity of the antigen detection confirmed constitutive, cytoplasmic/nuclear presence of HSPA1 in non-stressed keratinocytes in all epidermal layers, but not in the dermis (Scieglinska et al. 2011; Wang et al. 2011; Kleszczyński et al. 2015; Murase et al. 2016). Interestingly, keratinocytes in African Americans contained more HSPA1 than those in White Americans, what might implicate that HSPA1 in keratinocytes prevents the degradation of melanosomes after their transfer from melanocytes (Murase et al. 2016). Constitutive expression of the HSPA1 protein in epidermal keratinocytes was also found in rodents in vitro (Quenneville et al. 2002; Matsuda et al. 2010) and in vivo, albeit in contrast to humans preferentially in the upper epidermal layers (Laplante et al. 1998; Keagle et al. 2001; Souil et al. 2001).

The basal level (non-induced by stress) of HSPA1 expression in keratinocytes was suggested to depend on differentiation stage, but still it is unknown whether the protein is causally involved in the differentiation process. HSPA1 level was found relatively low in NHEK (3rd-passage) and HaCaT cells grown in low Ca^2+^ medium, but it was higher in high Ca^2+^ medium (Wakita et al. 1994; Orel et al. 1997). Interestingly, Ca^2+^-induced increase in HSPA1 expression could be suppressed by interaction of α-melanocyte stimulating hormone (αMSH) with its receptor present on keratinocytes (Orel et al. 1997).

Molecular mechanisms supporting constitutive expression of HSPA1 in normal keratinocytes are not known, and available data did not confirm the central role of HSF1 transcription factor. Although NHEK and HaCaT cells contain significant levels of HSF1 (Zhou et al. 1998; Nakamura et al. 2010), shRNA-mediated knockdown of HSF1 expression in HaCaT cells neither reduced HSPA1 expression, nor affected cell proliferation and viability (Nakamura et al. 2010). Additionally, it seems that some cell-specific mechanisms can control HSF1 functionality in skin cells. While overexpression of oncogenic RAS mutant (H-RasV12) in HaCaT cells induced nuclear translocation of transcriptionally competent HSF1 trimers, in contrast, repression of HSF1 activity could be observed in DFs (Zamkova et al. 2013). Based on the observation that basal level of HSPA1 was higher in keratinocytes from p53-deficient than from p53-proficient mice, one can assume that p53 may act as a negative regulator the HSPA1 gene expression in the epidermis (Quenneville et al. 2002).

### Expression of HSPA1 in keratinocytes after exposure to HS or UV stress treatment

Constitutive expression of HSPA1 in keratinocytes is thought to maintain their constant readiness to rapidly respond to various environmental stresses including heat and UV light. Though, the significant induction of the *HSPA1* gene expression in response to HS was demonstrated in NHEKs (Maytin et al. 1990, 1994; Schmidt-Rose et al. 1999; Parat et al. 1998; Hintzsche et al. 2012; Ramirez et al. 2015), HaCaT cells (Kao et al. 2016), human skin explants (Muramatsu et al. 1992), and in locally heated human skin areas in vivo (Wilson et al. 2000). HS-induced activation of the *HSPA1* gene expression, in contrast to its basal activity in non-stressed keratinocytes, was found to be HSF1-dependent (Zhou et al. 1998). A study of the global transcriptional response of human keratinocytes to HS (44 ^o^C, 40 min) revealed different kinetics of various HSPs induction. For example, the expression of *HSPA1* raised already at 4 h after HS, while the maximal levels of HSPB1 occurred 20 h later (Echchgadda et al. 2013). Because higher levels of HSPA1 were present in keratinocytes isolated from young (17–35 years old) than from elderly (65–90 years old) individuals, it seems that in keratinocytes, the stress response can be modulated by some age-related factors (Chinnathambi et al. 2008).

While the skin can be subjected to mild HS relatively often, its contact with a very high temperature occurs occasionally, either accidentally or during medical procedures. The expression of HSPA1 in NHEK can be induced even by a brief (1 s) severe HS (50–60 ^o^C) generated by laser pulse. Most NHEK survived temperature 60 ^o^C for as short a time as 1 s, or 55 ^o^C for 30 s, notably the consecutive short heat pulses induced thermotolerance (Bowman et al. 1997). Interestingly, HSPA1 level in epidermal keratinocytes can be also elevated by cold shock, as revealed by analysis of biopsies taken from human skin areas cooled to 15 ^o^C for 2 h (Holland et al. 1993). Similarly, HSPA1 expression also increased in human skin explants incubated overnight at 4 ^o^C (Jeanmaire et al. 2003).

One of the reasons for studying HSPA1 in skin was to assess its potential role in counteracting the harmful effects of phototoxicity generated by UV irradiation. It was shown that exposure of hairless mice (De la Coba et al. 2009) or keratinocytes (mouse, human) grown in standard 2D in vitro or 3D organotypic culture (Brunet and Giacomoni 1989; Jeanmaire et al. 2003; Perluigi et al. 2010; Yoshihisa et al. 2012; Wang et al. 2013; Kleszczyński et al. 2015) to UVB or UVA/UVB at various doses and experimental settings increased the levels of HSPA1 expression. However, Roh et al. (2008) observed no additional upregulation of HSPA1 after UVB or UVA irradiation in NHEK, A431 (epidermoid carcinoma) cells, while significant induction was found in DFs, in which basal level of HSPA1 is very low.

The levels of HSPA1 induction in UV-treated keratinocytes can be modulated by UV protective filters. In a study performed on hairless mice (Skr: hr-1), two potential photoprotector molecules (porfirin-334 and shinorine) isolated from red alga *Porphyra rosengurttii* were tested. UV irradiation caused rapid but transient increase of HSPA1 in keratinocytes and dermal cells in the skin of unprotected mice. In contrast, apical application of porfirin-334 plus shinorine resulted in delayed, gradual, and prolonged elevation of HSPA1 levels after UV exposure (De la Coba et al. 2009). The pattern of HSPA1 expression after UV irradiation can be also affected by psoralen, the drug frequently used in treatment of psoriasis and vitiligo. The study performed on skin explants taken from healthy volunteers shown that the combined treatment with UVA and methoxypsoralen induced translocation of HSPA1 into the nuclei of keratinocytes, while no such an effect was observed if explants were treated separately (Muramatsu et al. 1993).

Several lines of evidence point to elevated level of HSPA1, as to the key factor mediating adaptive response of keratinocytes to UV irradiation. This conviction comes partially from the studies demonstrating that keratinocytes in vitro (Maytin et al. 1993, 1994) and in vivo (Kane and Mayytin 1995; Trautinger et al. 1996) as well as A431 cancer cells in vitro when pretreated with HS become more resistant to UV irradiation (Trautinger et al. 1995b; Merwald et al. 2006). Similarly, keratinocytes resistance to UVB irradiation increased following treatment with HSPA1 inducers such as prostaglandin J2 (15dPGJ2, 1–2 ng/ml) (Merwald et al. 2006) or alkanin, an active constituent from the root extract of *Alkanna tinctoria* of the Boraginaceae family (Yoshihisa et al. 2012). Cytoprotective action of HSPA1 against UVB-induced toxicity and inflammatory response was demonstrated in studies performed using transgenic mice and murine PAM212 cells constitutively overexpressing the human HSPA1 protein (Matsuda et al. 2010). In contrast, targeting HSPA1 expression either with antisense oligonucleotides (Trautinger et al. 1995b) or by microinjection of anti-HSPA1 antibody (Bayerl and Jung 1999) sensitized keratinocytes to UVB-induced death. Interestingly, the study of Kleszczyński et al. (2015) shown that the need for protective activity of HSPA1 in UV-irradiated keratinocytes can be alleviated by melatonin, a hormone having strong antioxidative capacity. The authors found that melatonin can significantly reduce HSPA1 induction in full-thickness artificial skin irradiated with UV.

The strong evidence supporting cytoprotective activity of HSPA1 in epidermal keratinocytes was provided by transgenic mice models. While the appearance of the skin in wild-type (wt) and HSPA1 knock-out mice was similar, a massive dying of keratinocytes in the basal layer of epidermis was detected 12 and 24 h after the UVB irradiation (500 mJ/cm^2^) in HSPA1 knockout, but not in wt mice. At 24 h after irradiation, the entire epidermis of HSPA1 null mice was necrotic. The authors suggest that the lack of HSPA1 had a major negative impact of the DNA repair process (Kwon et al. 2002). Furthermore, keratinocytes of transgenic mice ectopically overexpressing the HSPA1 protein under the control of a constitutive β-actin promoter showed increased resistance to UV-induced apoptosis and contained decreased level of 8-OHdG and pyrimidine dimers, both approved markers of DNA damage (Matsuda et al. 2010). It has to be noted, however, that transient accumulation of HSPA1 in keratinocytes and dermal cells in response to UV irradiation did not render cells in irradiated skin areas resistant to UV-induced damage (De la Coba et al. 2009).

### Modulators of HSPA1 expression gene other than HS and UV irradiation

Keratinocytes can overexpress HSPA1 in response to multiple toxic factors. Among them are, e.g., Ni compounds (Carroll and Wood 2000), fluorine (Prado et al. 2011), zinc-chelator *N*,*N*,*N′*,*N′*-tetrakis(2-pyridinylmethyl)-1,2-ethanediamin (Parat et al. 1998), antibacterial zinc pyrithione, a DNA-damaging drug (Lamore et al. 2010), HgCl_2_ (Jeanmaire et al. 2003), ectoine, an osmoprotectant derived from *Halomonas elongata* (Buommino et al. 2005), SDS, a skin irritant (Niwa et al. 2009), prostaglandin Δ^12^-PGJ_2_ (Ikai et al. 1992), or sorbitol at concentration inducing osmotic stress (Garmyn et al. 2001). The increased levels of HSPA1 were also reported in keratinocytes stably transfected with HPV16 E6/E7 oncoproteins (Liao et al. 2005). As it was mentioned above, HSPA1 expression in the epidermis can be reduced by non-toxic drugs such as melatonin (Kleszczyński et al. 2015).

### HSPA1 expression in the skin exposed to thermal laser irradiation

In general, lasers through converting light into heat are used in dermatology in order to obtain selective photothermolysis of skin areas to initiate its remodeling. Which fragment of the skin is mostly affected depends on the length of laser light, targeted chromophore (essentially hemoglobin, water and melanin), beam power, and parameters of the treatment modality used. Non-ablative treatment induces thermolysis of dermis as well as lower epidermal strata leaving *stratum corneum* essentially intact, while ablative treatment damages the whole epidermis and the subcutaneous layer of the skin as well. The size of the wounded area depends of the methodology used. In fractional photothermolysis, multiple microscopic laser beams create vertical ablated channels, so called microthermal/microtreatment necrotic zones (MTZ) leaving undamaged tissues between them (rev in: Paasch 2016). The studies performed so far revealed that the level of HSPA1 increases in the epidermis surrounding the skin area damaged by thermolysis both in the case of ablative and non-ablative treatment. The maximal increment was observed between 4 h and 3 days post-treatment depending on the experimental schedule. Of note, no particular role of this effect in wound healing and re-epithelization was pointed out (Laubach et al. 2006; Hantash et al. 2007; Helbig and Paasch 2011; Sajjadi et al. 2013).

Results of studies on the effect of laser-mediated skin heating without causing thermolysis showed a possible involvement of HSPA1 in the re-epithelializaton process. In hairless rats, such a laser treatment increased the level of HspA1 in the irradiated skin (Souil et al. 2001), specifically in keratinocytes of *stratum spinosum* and *stratum basale*, and improved the process of wound healing (Capon et al. 2001). Further research in murine models, including HspA1 knockout mice, confirmed that laser-assisted pre-heating of the skin triggering HspA1 overproduction ameliorated the wound healing process (Wilmink et al. 2008; Makowski et al. 2012). Importantly, recent observation made in women 1 year after breast reduction showed that laser irradiation designed to trigger HSP synthesis in surgical incisions improved quality and cosmetic appearance of postsurgical scars (Casanova et al. 2017).

As a comment to the data mentioned above, one has to keep in mind that the heat shock response is a universal phenomenon, which takes place both in dermal and epidermal cells. Thus, without further in-depth studies, it is not possible to assess the extent to which both constitutive and laser-induced expression of HSPA1 in keratinocytes contributes to the healing of wounded skin. It has to be also added that so far, there is no data on whether keratinocytic HSPA1 has any role in the process known as photobiomodulation in which amelioration of wound healing results from non-thermal light treatment (Mosca et al. 2019). Such treatment known also as low-level light/laser therapy was shown to promote proliferation and maturation of keratinocytes (Sperandio et al. 2015); thus, involvement of HSPs cannot be excluded. We believe that the issue of laser- and/or light-induced modulation of the HSPs expression in keratinocytes needs further study since this problem appears important in the context of possible biostimulation in the treatment of chronic wounds.

### Extracellular HSPA1 (eHSPA1) as an immunomodulatory factor

Keratinocytes spontaneously release high levels of eHSPA1 to extracellular milieu. Although such a behavior is not unique to keratinocytes, these somatic cells can release much more eHSPA1 than any of the other ones, and the basal secretion rate can be raised by irradiation with UVB (Wang et al. 2011) or laser-diode (Sokolovskii et al. 2014). Secretion of eHSPA1 was also observed following treatment of HaCaT and A431 cells with U73122, an inhibitor of phospholipase C (Evdonin et al. 2004, 2006). Multiple studies specify eHSPA1 as a danger signal that induces immune responses. In this context, it is crucial that keratinocytes have an ability not only to secrete but also bind and internalize eHSPA1 and HSPA1-peptide complexes. The uptake of eHSPA1 into keratinocytes, the rate of which can be enhanced by some pro-inflammatory mediators (TNFα, IL-27, HMGB1), is mediated in an autocrine manner via low-density lipoprotein receptor-related protein-1 (LRP-1)/CD91 receptor (Wang et al. 2011). It is noteworthy that some reports denied presence of LRP-1/CD91 receptor on keratinocytes in healthy epidermis (Curry et al. 2003; Boyman et al. 2005). Because increased levels of eHSPA1 were found in cutaneous inflammatory pathologies such as psoriasis (Boehncke et al. 1994; Curry et al. 2003; Boyman et al. 2005; Gamal el Din et al. 2010), LE (Ghoreishi 2000; Mišunová et al. 2017; Jacquemin et al. 2017), and AD (Ghoreishi et al. 2000), this protein has been implicated in pathophysiology of these diseases.

Examination of human psoriatic lesion, as well as grafts of pre-psoriatic human skin (able to spontaneously develop into psoriatic lesions), onto immunocompromised mice revealed that HSPA1 accumulates in keratinocytes in the close vicinity of immune cells, in particular dendritic cells expressing the LRP-1/CD91 receptor. It was thus proposed that eHSPA1 released from psoriatic keratinocytes, via interacting with LRP-1/CD91 receptor present on dendritic cells, can activate pro-inflammatory responses (Boyman et al. 2005). The HSPA1-overexpressing keratinocytes were also detected in LE and vitiligo lesions close to the areas infiltrated with plasmocytoid dendritic cells. In this context, it is important to mention that exogenous recombinant HSPA1 can stimulate the cultured dendritic cells via binding to the Lox-1 receptor. This interaction was sufficient to stimulate the uptake the CpG type A oligonucleotides into dendritic cells and induce the production of INF-α. This cytokine in turn can activate expression of CXCL9/10 genes coding for chemokines attracting CD8(+) T cells (Jacquemin et al. 2017). Importantly, it has been recently found that topical application of a cream containing high concentration of inducible HSPA derived from alfalfa plant (*Medicago sativa*) onto murine skin reduced skin inflammation in experimental psoriatic-like lesions induced by imiquimol (Seifarth et al. 2018).

Findings showing that HSPA1 is located at the dermo-epidermal junction in the non-lesional skin of LE patients (Villalobos-Hurtado et al. 2003) lead to suggestion that HSPA1 itself can be an autoantigen and/or can participate in transferring SLE-related autoantigens to dermo-epidermal junctions. Recent studies showed that HSPA1 can be induced to significantly higher levels by UVB irradiation in keratinocytes from non-lesional skin of SLE patients than in keratinocytes from healthy donors. Therefore, it has been hypothesized that severe environmental stressors (e.g. UVB) via increased production and release of eHSPA1 from keratinocytes may have profound effect on SLE development in predisposed individuals (Jacquemin et al. 2017). It is likely that eHSPA1 as a danger signal could activate dendritic cells to produce type I interferon, a cytokine involved in various cutaneous inflammatory and autoimmune disorders. Interestingly, an association between the polymorphic changes in HSPA1 locus with predisposition to SLE was reported (Fürnrohr et al. 2010).

One of pro-inflammatory factors involved in the development of AD is thymic stromal lymphopoietin (TSLP), an IL-7-like chemokine/cytokine produced by keratinocytes. The release of TSLP was found to be suppressed in HaCaT cells exposed to HS or in keratinocytes incubated with exogenous recombinant HSPA1. This inhibitory action of HSPA1 on TSLP secretion was mediated by inhibition of NF-κB activation. These observations suggested that exaggerated secretion of eHSPA1 from keratinocytes could be a potential component of AD therapy (Kao et al. 2016).

## Essentials about the HSPA2 protein

The HSPA2 chaperone of HSPA family was first identified in rats and mice as being abundantly expressed in pachytene spermatocytes (Zakeri et al. 1988; Krawczyk et al. 1988; Wisniewski et al. 1990) and subsequently described as a testis-specific protein critical for mouse spermatogenesis (Dix et al. 1996). The human *HSPA2* gene shows high similarity (approx. 92% and 98% at nucleotide and amino acids levels, respectively) to its rodent counterparts (Bonnycastle et al. 1994). Transcriptional and IHC analyses revealed that HSPA2 in rodents and more prominently in humans, beside male meiotic cells, is also expressed in certain somatic tissues (Bonnycastle et al. 1994; Scieglinska et al. 1997; Vydra et al. 2009; Scieglinska et al. 2011). High levels of HSPA2 in humans were confined among others to brain and stratified epithelia covering skin and esophagus, as well as pseudostratified epithelia lining bronchus (Scieglinska et al. 2011). Moreover, HSPA2 was also detected in numerous primary malignancies (Scieglinska et al. 2014). However, a significance of HSPA2 for cancer diagnosis, prognosis, or treatment has to be determined.

The mechanisms controlling the human *HSPA2* gene expression are poorly understood. It is clear that *HSPA2* is a representative of non-heat-inducible *HSPA* genes (for review see: Scieglinska and Krawczyk 2015); its transcription is under direct control of hypoxia inducible factor 1 (HIF-1) (Huang et al. 2009; Habryka et al. 2015), through binding to the hypoxia response element in the *HSPA2* gene promoter (Habryka et al. 2015). Interestingly, HIF-1 in relation to the *HSPA2* gene seems to act as a transcriptional activator in cancer cells, but as a repressor in keratinocytes (Habryka et al. 2015). The role of HSPA2 is best recognized in the context of its contribution to spermatogenesis and fertilization, but poorly characterized in somatic cells (reviewed in Nixon et al. 2017; Scieglinska and Krawczyk 2015). Up to now, only general chaperoning and cytoprotective properties of HSPA2 were demonstrated in vitro in cells overexpressing the human protein (Hageman et al. 2011; Filipczak et al. 2012).

### Expression and function of HSPA2 in epidermal keratinocytes

IHC studies revealed that HSPA2 is present in the human skin in the cytoplasm and nuclei of keratinocytes located selectively in the basal layer (Scieglinska et al. 2011; Gogler-Pigłowska et al. 2018). Thus, HSPA2 in the epidermis showed the unique expression pattern, entirely distinct from that described previously for HSPB1 or HSPA1. Fluorescence activated cell sorting (FACS) of live NHEK according to their differentiation status followed by semi-quantitative analysis of mRNAs encoding HSPA2 and differentiation markers showed lowest level of HSPA2 transcripts in NHEK fraction enriched in quiescent progenitors/stem cells (Gogler-Pigłowska et al. 2018).

Results of shRNA-mediated knockdown of HSPA2 expression in HaCaT cells suggest that this chaperone neither protects the cells against HS-induced cytotoxicity, nor supports their proliferation. HSPA2-deficient HaCaT cells showed, however, reduced clone-forming ability, delay in spreading onto collagen IV, and reduced adhesiveness to fibronectin- or collagen IV-coated surfaces. These features of HSPA2-deficient keratinocytes classify them as representing more mature phenotype. The results of RHE model analysis led us to conclude that HSPA2 can contribute to mechanisms which restrain keratinocytes from premature entry into the terminal differentiation process (Gogler-Pigłowska et al. 2018). These observations suggest that HSPA2 can participate in controlling keratinocyte fate decision in the basal layer of epidermis.

Results of several studies pointed out that expression patterns (and possibly some functions) of HSPA2 in murine and human skin can be distinct. Analysis of transgenic mice bearing GFP reporter gene under the control of the promoter of the rat *HSPA2* gene showed the presence of the reporter protein in cells of the hair bulbs (Vydra et al. 2009). In another study, the endogenous HSPA2 was located in suprabasal cells of the inner root sheath (Gunnarsson et al. 2016). Taking into account that skin alterations were not noticed in young HSPA2 knockout mice (Dix et al. 1996), one might speculate that the influence of HSPA2 on the maintenance of epidermal homeostasis in murine skin may be subtle or may change/enhance with aging.

## Essentials about HSPC1 (HSP90α) and HSPC3 (HSP90β) proteins

HSPC1 and HSPC3 are chaperone proteins of 90 kDa molecular weight, which together with HSPC4 (GRP94) and HSPC5 (TRAP-1) proteins group into the HSPC (HSP90) family. In mammals, HSPC1 and HSPC3 are two homologous isoforms sharing approx. 86% of aa sequence similarity; they are encoded by *HSPC1* (*HSP90AA1*), and *HSPC3* (*HSP90AB1*) genes, respectively (Sreedhar et al. 2004). The basal levels of both isoforms may reach up to 2% of total cytosolic proteins and can be doubled under cellular stress due to induction of the HSPC1 expression. Under physiological conditions, HSPC3 is expressed constitutively at significantly higher basal level than the stress-inducible HSPC1 (Zuehlke et al. 2005). HSPC1 and HSPC3 isoforms as multifunctional chaperones are engaged in activation, conformational stabilization, and final maturation of multiple cellular proteins; thus, it performs key functions in the maintenance of cellular proteostasis, being considered as a hub in the network of molecular chaperones (Eckl and Richter 2013; Röhl et al. 2013; Karagöz and Rudiger 2015). So far, about 700 substrates of HSPC (referred to as its “clients”) have been characterized, which comprise proteins of various functional groups such as transcription factors, kinases, ubiquitin ligases, receptors, and others (Miyata et al. 2013). Following the discovery of the first effective HSPC inhibitor, namely antibiotic geldanamycin (GA), the development and testing in clinical trials of multiple HSPC-targeting drugs has been started (Tatokoro et al. 2015).

### Expression pattern and function of HSPC in keratinocytes

Compared to other organs, the endogenous expression of HSPC in the skin is rather low, and the protein level is lower in the epidermis than in the dermis (Wilson et al. 2000; Kakeda et al. 2014; Tukaj et al. 2013). The usage of isoform-specific antibodies revealed that HSPC1 is present preferentially in the epidermis, whereas HSPC3 in the dermis (Cheng et al. 2008). Increased expression of HSPC, mainly the HSPC1 isoform, was observed in psoriatic keratinocytes (Boehncke et al. 1994; Kakeda et al. 2014). Elevated expression of HSPC was also found in epidermal keratinocytes in perilesional skin areas in patients with bullous pemphigoid (BP) (Tukaj et al. 2013).

Under physiological conditions, HSPC1 is located in the cytoplasm and partially in the nuclei of keratinocytes, while a minor fraction of the protein is present extracellularly. However, HSPC1 can be efficiently secreted in a HIF-1α-dependent manner from keratinocytes in hypoxic microenvironment of wounded skin (Woodley et al. 2009). Importantly, hypoxia promotes migration of human keratinocytes (O'Toole et al. 1997) and DFs (Li et al. 2007), a critical phenomenon in re-epithelialization process during skin wound healing. Intracellular level of HSPC and the rate of its release from keratinocytes are sensitive to cytokines present in the serum, e.g., to transforming growth factor-alpha (TGFα), which causes secretion of HSPC1 via exosome pathway (Cheng et al. 2008). A study performed on HaCaT cells exposed to serum of bullous pemphigoid (BP) patients indicated that the secretion of HSPC from keratinocytes can be inhibited by BP autoantibodies, but not by control IgG from healthy donors (Tukaj et al. 2013). Importantly, blockade of HSPC activity by geldanamycin derivative 17-DMAG reverted BP autoantibodies-mediated pro-inflammatory response of keratinocytes (Tukaj et al. 2014). Since contribution of HSPC to other autoimmune blistering dermatoses including epidermolysis bullosa acquisita and possibly dermatitis herpetiformis was also found, more universal roles of HSPC as a pathophysiological factor and potential target for treatment of autoimmune BP diseases have been suggested (Tukaj et al. 2015).

A number of studies were aimed at determining the role of HSPC in regulating keratinocytes migration during wound healing. Skin injury (wound or burn) initiates the complex, multistep healing process that includes inflammation, tissue formation through re-epithelialization, angiogenesis and matrix protein production, and finally tissue remodeling. The closure of human wounds is largely mediated by re-epithelialization, a process accompanied by acquisition of highly active keratinocyte phenotype, characterized by continuous proliferation and high migratory properties (Rittie 2016). The pool of extracellular HSPC1 (eHSPC1) plays an essential role in switching to this phenotype (Woodley et al. 2009), while HSPC3 was found important for keratinocytes viability (Zou et al. 2017). A model explaining the role of HSPC1 in the re-epithelialization postulates that wounding, via hypoxia and HIF-1α-dependent signaling, stimulates secretion of eHSPC1 from laterally migrating keratinocytes. Subsequent binding of eHSPC1 to low-density lipoprotein receptor-related protein-1 (LRP-1) receptor present in keratinocytes stimulates, in an autocrine loop, keratinocyte migration for re-epithelialization (Woodley et al. 2009, 2015). Further studies showed that the LRP-1/CD91-mediated pro-migratory signaling is critically dependent on AKT1 and AKT2 activity (Tsen et al. 2013). eHSPC1 can also participate in inducing migration of DFs and microvascular endothelial cells (HDMECs), thus enabling fibroplasia and neo-vascularization (Li et al. 2007). Although the detailed mechanism has not been established yet, it is known that eHSPC1, after reaching a critical threshold concentration, can override the inhibitory effect of TGFβ3 on DFs and HDMECs migration (Cheng et al. 2008).

The role of eHSPC1 in stimulating keratinocytes migration was also demonstrated on murine (Zhang et al. 2014) and pig models (O’Brien et al. 2014). Studies in nude mice showed that repeated local application of human recombinant HSPC1 onto the wounded dorsal region accelerated wound closure at approx. 30% (Li et al. 2007). Consistently, topical application of HSPC1 in the early stage of a deep second-degree burn wounds in murine skin led to reduced inflammation and increased tissue granulation, with a concomitant reduction in the size of the wound (Zhang et al. 2014). The examination of deletion mutants of HSPC1 provides evidence that the non-chaperone function of this protein is essential during wound closure. The pro-motility action could be exerted by both the short 115 aa fragment of HSPC1 molecule (F-5 peptide) and HSPC1-Δ variant deficient in 232-amino acid from the carboxyl terminus, which was unable to dimerize and function as an intracellular chaperone (Cheng et al. 2011; O’Brien et al. 2014; Bhatia et al. 2016, 2018). When it comes to F-5 peptide, its application to wounded skin significantly improved healing of acute and diabetic wound in mice (Cheng et al. 2011), as well as in diabetic pig (O’Brien et al. 2014). Topical application of F-5 also effectively promoted healing of experimental burn wounds in pigs (Bhatia et al. 2016). Moreover, transfection of the human umbilical cord mesenchymal stem cells (hUC-MSCs) with the construct encoding F-5 improved survival of skin flaps subjected to ischemia-reperfusion experiment (Leng et al. 2017). In respect to transgenic mice bearing the chaperone-defective HSPC1-Δ mutant, the rate of wound closure was similar as in the wild-type HSPC1 mice. Importantly, selective inhibition of the eHSPC1-Δ protein by a monoclonal antibody disturbed normal wound closure in both wild-type HSPC1 and HSPC1-Δ mice (Bhatia et al. 2018). Pro-motility action of eHSPC1 can be considered a universal property of this chaperone since it was found critical for the invasiveness of cancer cells (Wong and Jay 2016). A more extensive information on the role of eHSPC1 in wound healing can be found in the recent review article (Guo et al. 2017).

A potential role of HSPC in regulating epidermal homeostasis is suggested by only a limited number of data. HSPC inhibition by 17-AAG (a geldanamycin derivative) in HaCaT cells blocked calcium-induced differentiation. This observation suggests that HSPC can contribute to early stages of keratinocyte differentiation (Miyoshi et al. 2012). The expression of HSPC was also found sensitive to disruption of skin barrier caused by tape stripping of *stratumcorneum* (Dickel et al. 2010). The increased level of HSPC was also suggested to promote development of UV-B-induced cutaneous squamous cell carcinogenesis (Singh et al. 2015). Analysis of nude mice chronically (3 times per week for 25 weeks) exposed to UV light showed that local application of 17-AAG before each irradiation resulted in abnormalities such as high epidermal hyperplasia. At molecular level, this response was associated with reduced interaction of HSPC3 with PKCε, and decreased expression of pSTAT3 and pAKT. Epidermal abnormalities were also observed during embryogenesis in mutant mice lacking p23, a HSPC co-chaperone (Madon-Simon et al. 2017).

## Summary and final conclusions

Epidermis and keratinocytes are highly valuable models for investigating the complexity in expression and function of various HSPs. The current knowledge of molecular and cellular mechanisms underlying the processes of epidermal stratification and re-epithelialization gives opportunity to expand our understanding of HSPs involvement in the maintenance and restoration of homeostasis in adult tissue. The spatial and functional stratification of keratinocytes in the epidermis also facilitates the study of cytoprotective mechanisms in the context of tissue predisposed to frequent contact with multiple environmental stressors. Technical improvements, in particular development of three-dimensional human skin equivalents for in vitro assays, in combination with possibility of genetic manipulations of cutaneous cells enable the investigation of a particular gene in tissue formation, epidermal cells differentiation, phenotypic reprogramming, and death of keratinocytes.

In spite of above-mentioned achievements, knowledge of the importance of HSPs in epidermal differentiation, wound healing, development of keratinocytic carcinoma, and non-cancerous skin diseases, as well as in cytoprotection against harmful environmental factors, is still far from being satisfactory. One of highly relevant issues, which is still not sufficiently explained in the functional context, is stratum-specific expression of HSPB1 and HSPA2 chaperones, for which no convincing confirmation of their cytoprotective role in keratinocytes has been provided. Different spatial localization of these two proteins in the epidermis seems to reflect their distinct contribution to keratinocyte differentiation. HSPA2 was shown to prevent the basal keratinocytes from premature entry into terminal differentiation process, while HSPB1 plays a role during the final steps of keratinization and formation of epidermal barrier. There is no doubt that intensive work has to be done in order to fully determine function of these chaperone proteins in the biology of epidermis.

In contrast, stress-inducible HSPA1 is nonselective for epidermal strata, shows no relationship with keratinocyte differentiation, but it can ensure quick and effective protection against various environmental stressors. Taking into account that HSPA1 can be secreted from keratinocytes, the further research on the impact of eHSPA1 in pathophysiology of inflammatory skin disorders deserves special attention.

With regard to HSPC proteins, it seems particularly important to notice differential expression of HSPC1 and HSPC3 isoforms in the normal and pathological skin. Although there are some hints that HSPC can participate in early steps of keratinocyte differentiation, the most firmly accepted knowledge concerns the regulatory role of eHSPC1 in keratinocyte and DFs migration during wound healing.

In summary, the studies performed so far indicate that HSPB1, HSPA1, HSPA2, and HSPC in keratinocytes contribute both to the maintenance of epidermal homeostasis as well as to skin pathophysiology, although the molecular mechanisms behind these functions are poorly understood. Thus, it seems justified to intensify studies aimed at determining how changes in HSPs expression, alterations in their posttranslational modifications, or secretion rate would influence the skin homeostasis and impact on etiopathogenesis of cutaneous diseases. Such knowledge would be highly valuable for better understanding and treatment of skin diseases and wound healing.
